# Neuro-impressions: interpreting the nature of human creativity

**DOI:** 10.3389/fnhum.2012.00282

**Published:** 2012-10-12

**Authors:** Todd Lael Siler

**Affiliations:** ArtScience® PublicationsDenver, CO, USA

**Keywords:** ArtScience, creativity, discovery, invention, innovation

## Abstract

Understanding the creative process is essential for realizing human potential. Over the past four decades, the author has explored this subject through his brain-inspired drawings, paintings, symbolic sculptures, and experimental art installations that present myriad impressions of human creativity. These impressionistic artworks interpret rather than illustrate the complexities of the creative process. They draw insights from empirical studies that correlate how human beings create, learn, remember, innovate, and communicate. In addition to offering fresh aesthetic experiences, this metaphorical art raises fundamental questions concerning the deep connections between the brain and its creations. The author describes his artworks as embodiments of everyday observations about the neuropsychology of creativity, and its all-purpose applications for stimulating and accelerating innovation.

## Introduction

I make art about the brain, and learn about the brain through art. This remains my lifelong passion and challenge: discovering how the human brain constantly learns about itself by studying its countless creations. That's the central theme of my artwork, which considers how the brain is connected to all of its creations in every way imaginable, and how brain mechanisms form and shape our lives and future.

The eclectic aesthetics of my artworks reflect the broadest definition of Art, which encompasses **A**ll **r**epresentations of **t**hought. From my perspective, Art embraces all expressions and manifestations of creativity, embodying the collective work of human nervous systems and everything our minds make (McCulloch, 1988; Siler, 1993).

We tend to experience things by how we define them. When we encounter a work of art whose subject matter is neuroscience, we expect to see copious images of recognizable brain matter. It rarely occurs to us that all the creations of the mind we encounter daily (from houses to cities) bear little resemblance to the brain. And yet, these things reflect the handiwork of the human brain. How exactly, no one knows for certain. But it's one of the most exciting promises and prospects of neuroscience: to know how (Pinker, 2009). And, learn how to boldly think beyond the categories of our compartmentalized knowledge, sparking important innovations.

## Impressionism meets neural art

As the title of this article implies my art is mostly impressionistic. Meaning, it shares certain qualities of ambiguity and abstraction visible in Modern and Post-modern Art. It also shares a common wellspring of inspirations that connect my artistic interpretations of nature with Claude Monet's “Les Nympheas” (Water-Lilies); these visceral murals fill two oval-shaped, womb-like rooms at the Musée de l'Orangerieis in Paris (Tucker, 1998). The paintings grew in Monet's imagination for thirty years, well after this practitioner of *en plein air* (“in the open air”) painting had planted thousands of water lily bulbs, which evolved into the elegant pond he painted, studied and maintained like an outdoor lab.

Monet's impressionistic art appeals to my Neural Art, as it connects us to the world within and around us. Moreover, it inspired me to plant in my paintings all sorts of brain-related questions about how we perceive and understand the world; literally, I collaged my perceptions and concepts on my canvases, and cultivated these conceptual plantings over many years. Some seeds grew into these colorful depictions of human neurons shown here. They are meant to evoke images of a different, yet related, “garden of the mind” that Monet painted at Giverny.

The fundamental questions I have picked to explore are not the garden variety type, even though they are quite universal. For example, how is the human brain connected to nature, and how is nature connected to everything the brain creates? Specifically, how do the details of nature *detail* the nature of the brain?

Interpreting these open-ended questions has yielded a cornucopia of art forms that document my impressions of the creative process (Siler, 1995). These artworks share an aesthetic kinship with other contemporary visual artists who make tangible *the intangible* aspects of creativity, as well (Bailly, 1982; Kriesche, 1985; Arakawa and Gins, 1991). In effect, they are manifestations of “metacognition,” a term used in the field of education and cognitive neuroscience to describe the process of “knowing about knowing” and the practice of questioning our “cognitions about cognition” (Metcalfe and Shimamura, 1994).

## The fine art of thought

**Figure 1:**
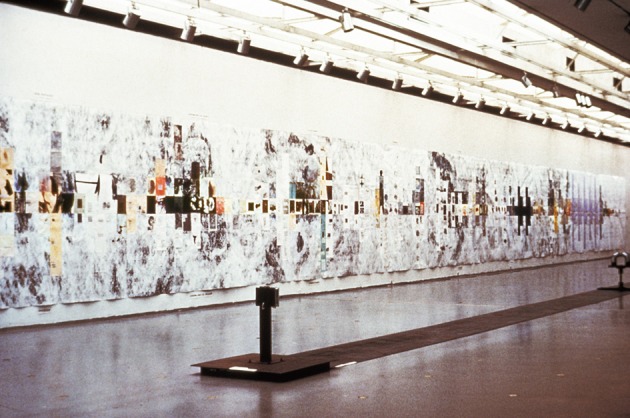
**“Thought-Assemblies,” 1979–1982.** Installation view at Musee D'Art Moderne de la Ville de Paris, France, 1982. Mixed media on synthetic paper and canvas, 9 × 127 ft. “Thought-Assemblies” details a process of incubating ideas and percolating on the possibilities of their actualization. This symbolic artwork draws on my formal studies of neuropsychology, which helped inform my visualizations of the creative process.

**Figure 2:**
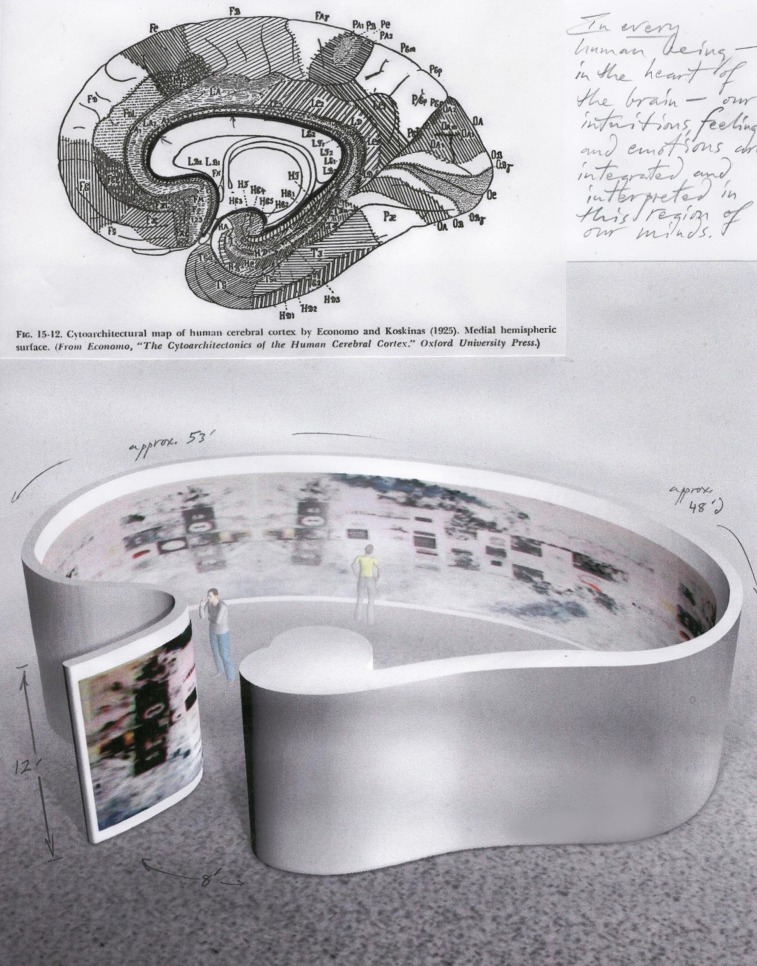
**“The Organizing Principle for *Thought-Assemblies*” (1979–1982).** Ink on paper, 10.5 × 8.5 inches.

**Figure 3:**
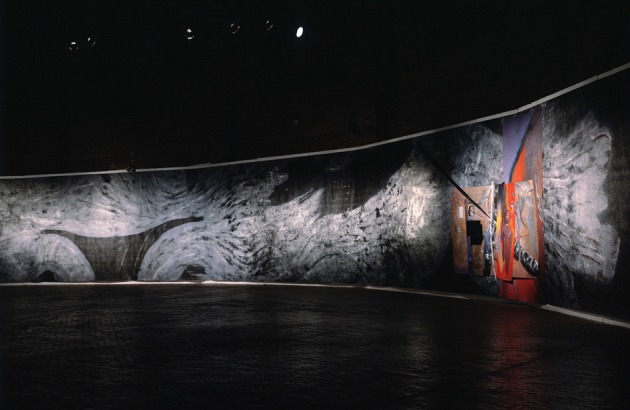
**“The Brain Theater of Mental Imagery” (1983).** Mixed mediums on spunbonded synthetic canvas, 12 × 100 ft., with mounted paintings and white light hologram, Installation view: Boston Center for the Arts, 1990.

The brain-inspired artworks of mine highlighted here (Figures 1–3) evolved from my graduate studies at MIT's Center for Advanced Visual Studies in 1979. At CAVS, I had the opportunity to freely explore a wide range of interrelated fundamental questions that focused on some deep connections between nuclear physics and neurophysiology. I expressed these connections metaphors, physical analogies and visual suppositions (Siler, 1981), following a path of creative inquiry cut by artists and scientists of the Italian Renaissance—most notably the quintessential ArtScientist, Leonardo da Vinci. As the thousands of pages of his Codices show, Leonardo spent his lifetime searching nature's connections, many of which focused on understanding the functional architecture of the brain (MacCurdy, 1938). DaVinci's search helped spur the collaborative efforts today in human neuroscience, in which teams of researchers systematically correlate the cause-and-effects of neural events aided by non-invasive medical imaging tools.

The neurophysiologist Eberhard Fetz eloquently summed up these ongoing efforts to grasp the the great unknowns of the brain that challenge our collective ingenuity, writing: “there is a largely unexplored area of brain function as itself a subject for artistic representation. The neural networks in our brains effortlessly perform common miracles of perceiving the world, controlling volitional movements and performing higher functions like speech and thought. These cognitive functions are all produced by complex patterns of neural activity, but how mental events emerge from material mechanisms remains an enduring mystery” (Fetz, 2012).

Ultimately, human development hinges on “understanding neurons “…” these aesthetic elementary microchips of the brain” (Segev, 2011), and understanding that our collective future rests on how wisely and ingeniously we apply our neural knowledge. To this way, a world of inquisitive minds seek insights into the symbolic languages of neurons, in the same visionary way that Pythagoras understood this reality: “mathematics is the nature of language,” like symbolisms is the language of nature. Naturally, we are all symbol-making creatures.

## Visualizing the nature of human creativity

As a generalist studying the human brain everyday, I use the fine arts (Figures 1–3) as instruments for hypothesizing and investigating the actions of neural systems that form, shape and influence every facet of our lives (Siler, 1988). The paintings interpret how our thoughts, feelings, actions and behaviors maybe traced to various neural mechanisms with the understanding that “correlation is not causation.”

In responding to my open-ended questions, I created one sprawling visual knowledge map that looks as long and complicated as a linear high-energy accelerator! This impressionistic artwork, titled “Thought-Assemblies” (Figures 1 and 2), interprets the interconnected process of creative and critical thinking. It poses these interrelated basic questions for everyone to ponder: How do the mechanisms of thought (nerve cell-assemblies and interactions) influence the contents of thought? Are action potentials, which relay information over long distances and synaptic potentials, which integrate information over short distances the signaling devices that change the meanings of our thoughts, feelings, and actions? (Siler, 1987). Are “thought-assemblies”—related patterns of mental activity or an association of ideas—the creations of “cell-assemblies?” What is a thought? A thing, or a product of some thing? A process, or something intangible? (James, 1890; Eccles, 1970).

“Thought-Assemblies” served as the visual component of my MIT dissertation, *Architectonics of Thought: A Symbolic Model of Neuropsychological Processes* in Interdisciplinary Studies in Psychology and Art. The artwork presents an alternative perspective on the neuropsychology of creativity—one that connects all acts of creating, discovering, inventing, innovating, collaborative learning, and problem solving (Siler, 1986). Moreover, it intimates how nature may be one interconnected creative process with countless manifestations.

The conceptual framework for this artwork builds on the work of the 20th century Canadian behavioral psychologist Donald O. Hebb's theory of cell-assemblies, which describes how neurons connect with one another to form groups of neuronal connections that fire together in various acts of learning (Doidge, 2007). He also noted that “thought must be known as theoretically as a chemist knows the atom” (Hebb, 1949).

The overall pattern of “Thought-Assemblies” resembles a giant EEG recording, suggesting that the mental states (e.g., varying degrees of alertness and levels of consciousness) are closely correlated with the shape of the EEG (its frequency and amplitude). These states of mind are represented in the virtual mental representations that I have collaged and mounted on a sensual synthetic paper. The mosaic of mental imagery documents ephemeral flashes of creative thinking as I envision them occurring within the real and virtual worlds of the mind. The apparent linearity of this artwork belies the non-linear, stochastic process of creativity (Siler, 1985).

Physically, this *thoughtform* is the size of a 12-story building turned on its side (see Figure 1). The “windows” of this virtual building are comprised of 515 pictures of mental representations organized in a seemingly orderly way. The artwork was meant to envelop its viewers, making them part of the art. In this way, I aimed to could show what's on my mind and they could read my thoughts, absorbing the concepts and contemplating the hypotheses. Some of my installation drawings envision this artwork stretching for miles. Other drawings show it shrunk to the tiny scale of a Very Large Storage Integration (VLSI) computer chip that could fit on your pinky's fingertip. Even that tiny scale may be too large, especially when viewed on the nanoscale (10^−9^m), where “size does matter”; in particular, it matters to our understanding of the hierarchy of influences at work in everything that is composed from the bottom up: “from the atom to clusters of atoms to nanomaterials to materials (Mendeleev, 1901); all exhibit different behaviors that are not just relevant to their different physical dimensions” (Ozin et al., 2009). That includes the human nervous system and all other forms of organic material.

“Thought-Assemblies” can be configured on curved or wavy walls, as shown in Figure 2. That particular wall is derived from the arc of the cingulate gyrus, which is part of the Limbic system. This region marks the “heart” of the brain (thalamas), where non-specific thalamic projections (Nauta and Whitlock, 1954) link higher and lower brain functions that directly influence our thoughts-feelings-and-actions (Chorover and Chorover, 1982). Within this region, I hypothesize, intuitions, insights, eurekas, and other emotionally-charged feelings occur, signaling the state of flow (Csikszentmihalyi, 1996) and inducing the simple pleasures of memorable aesthetic experiences that are processed by higher order cerebral systems (Siler, 1986; Cowley and Underwood, 1998, June 15; Damasio, 2000; Hesselink, 2011). Overall, it searches the neuropsychology of the brain that inspired its design and composed its contents, which encompass everything from poems on nature to studies of neuronal architecture.

## Glimpsing a future shaped by understanding creativity

Creativity remains an inexhaustible subject that is relevant to all aspects of human development, interactivity, and culture (Koestler, 1964; Root-Bernstein, 1985; Sternberg, 1988; Siler, 1997; Epstein, 1999). This subject is linked to and riddled by many of nature's deepest mysteries, among them: complexity, connectivity, and chaos. Understanding these phenomena and their relationship is the wonderful challenge of transdisciplinary thinkers, or ArtScientists, who sense that piecing together the great puzzle of creativity entails integrating all human knowledge (Root-Bernstein et al., 2011).

“The Brain Theater of Mental Imagery” (Figure 3) offers one unique environment for seeking and seeing some of the most puzzling connections that link neural mechanisms. In approaching this work with an open mind and liberated imagination, you are likely to glean how it unites all the elements of its creation, just as the human brain does (Siler, 1990).

Standing a few feet from this painting, you notice these neural-like networks or ganglia in the gray matter. These vigorously textured reliefs, created by layers of paint, reveal the unique printing and painting process that generated this giant, continuous monotype. It was created by an imaging invention of mine, which MIT patented with me in the early 1980s. One intriguing detail about this artwork and invention is the fact that it was inspired by some Golgi-stained neurons that Dr. Walle Nauta showed me along with his exquisite neuroanatomical drawings that are every bit as elegant as Santiago Ramon y Cajal's wondrous renderings (Cajal, 1899). These works show a similar “creative aesthetic” that unites the complementary sensibilities of the arts and sciences (Bronowski, 1956; Curtin, 1982; Root-Bernstein, 1996).

The artworks I have touched on here convey one overarching impression of our artistic-scientific-mathematical portraits of the human brain: all fall short of fully describing our collaborative minds' potentially limitless capabilities (Siler, 2011). And that's a good thing, as it suggests there are *surmountable* opportunities for developing useful scientific generalizations of brain dynamics applied to the advancement of humankind—rather than “insurmountable opportunities,” to echo the cautionary words of venture capitalists who must invest in these developments that invariably shape our future.

My art aims to challenge our concepts of limits (Medawar, 1984) by engaging and expanding our sense of wonderment (Weisskopf, 1979). “Wisdom begins with wonder,” Socrates said. And wonder propels and critiques our scientific pursuits of the truth (Morrison and Morrison, 1984), while heightening our awareness of our creative potential.

### Conflict of interest statement

The author declares that the research was conducted in the absence of any commercial or financial relationships that could be construed as a potential conflict of interest.
